# Type I Interferons in Bacterial Infections: A Balancing Act

**DOI:** 10.3389/fimmu.2016.00652

**Published:** 2016-12-26

**Authors:** Pavel Kovarik, Virginia Castiglia, Masa Ivin, Florian Ebner

**Affiliations:** ^1^Max F. Perutz Laboratories, University of Vienna, Vienna, Austria

**Keywords:** type I interferon, bacterial infection, cytokines, chemokines, innate immunity, resilience to infections, immunomodulation, immunosuppression

## Abstract

Defense against bacterial infections requires activation of the immune response as well as timely reestablishment of tissue and immune homeostasis. Instauration of homeostasis is critical for tissue regeneration, wound healing, and host recovery. Recent studies revealed that severe infectious diseases frequently result from failures in homeostatic processes rather than from inefficient pathogen eradication. Type I interferons (IFN) appear to play a key role in such processes. Remarkably, the involvement of type I IFNs in the regulation of immune and tissue homeostasis upon bacterial insult may have beneficial or detrimental consequences for the host. The reasons for such ambivalent function of type I IFNs are not understood. The disparate effects of type I IFNs on bacterial infections are in marked contrast to their well-established protective roles in most viral infections. In this review, we will focus on type I IFN effector mechanisms which balance processes involved in immune and tissue homeostasis during specific bacterial infections and highlight the most important missing links in our understanding of type I IFN functions.

## Introduction

**Figure 1:**
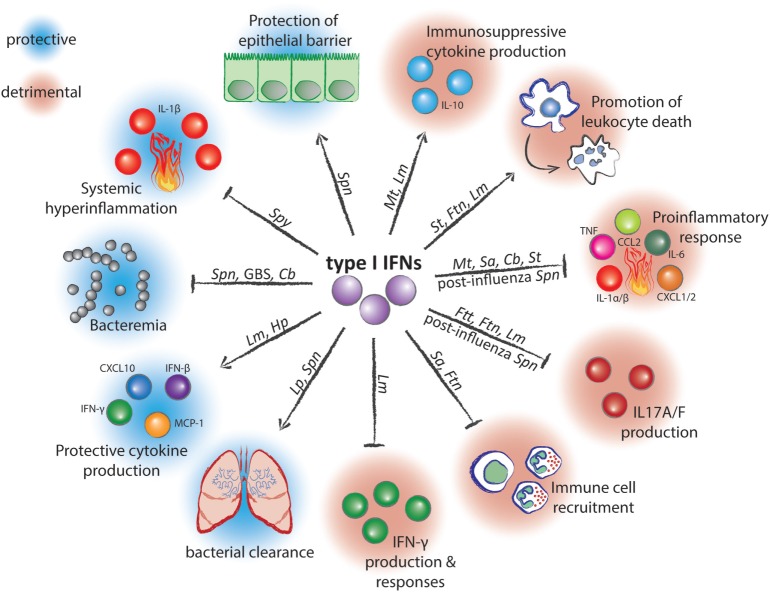
**Mechanisms of action and effects of type I interferons (IFNs) during infection with bacterial pathogens**. Arrow-headed lines represent stimulation and bar-headed lines represent inhibition by type I IFNs. Pathogen abbreviations: *Spn, Streptococcus pneumoniae*; *Spy, Streptococcus pyogenes*; GBS, Group B Streptococcus; *Cb, Coxiella burnetii*; *Lm, Listeria monocytogenes*; *Hp, Helicobacter pylori*; *Lp, Legionella pneumophilia*; *Sa, Staphylococcus aureus*; *Ftn, Francisella tularensis* subspecies novicida; *Ftt, Francisella tularensis* subspecies tularensis; *Mt, Mycobacterium tuberculosis*; *St, Salmonella enterica* serovar Typhimurium.

Successful defense against pathogens requires both, the eradication of the infectious agent by the immune system as well as tissue protection against the damaging effects of the immune response. Increasing evidence indicates that many if not most infectious diseases result from insufficient resilience, i.e., from a failure of the infected host to repair and regenerate destroyed tissues, rather than from inefficient pathogen clearance (1–4). Mechanisms which preserve the integrity of host tissues during the intensive inflammatory response against the pathogen remain incompletely understood. Recent studies established that successful tissue protection during infection requires systems which balance the immune response as well as mechanisms which restore tissue homeostasis. These mechanisms are often interdependent and result from messengers like growth factors, cytokines, or lipids produced by immune cells (1–4). Examples include the anti-inflammatory cytokine IL-10, the tissue regeneration promoting IL-22 and amphiregulin, the tissue remodeler TGF-β, or the pro-resolving lipid lipoxin (5–8). Remarkably, several recent studies demonstrated that type I interferons (IFNs) can also act as critical resilience-promoting cytokines during infections with several streptococcal species (9–11). Such protective functions are in marked contrast to detrimental effects of type I IFN during infections with many other bacterial species (12, 13). The reasons for the ambivalent roles of type I IFNs in bacterial infections remain poorly understood. However, it appears that the ability of type I IFNs to both suppress and stimulate immune responses is of critical importance (Table 1; Figure 1). This review focuses on the role of type I IFNs in balancing pro- and anti-inflammatory processes as well as cell survival and cell death programs during antibacterial defense and discusses how these effects determine the outcome of an infection.

**Table 1:** **Effects of type I interferons (IFN) signaling in bacterial infections**.

Pathogen	Type of bacteria	Route of infection	Model of infection	Effect of type I IFN signaling	Mechanism	Reference
*Streptococcus pneumoniae*	Gram+, extracell	Intranasal; intratracheal	Model of lung infection	**Protective**	Protection against epithelial barrier damage	(10, 11, 14)

*Streptococcus pyogenes*	Gram+, extracell	Subcutaneous	Model of invasive cellulitis	**Protective**	Prevention of IL-1β-driven systemic hyperinflammation	(9, 15)

Group B streptococcus	Gram+, extracell	Intraperitoneal (adults); subcutaneous (neonates)	Model of systemic infection/sepsis	**Protective**	Protection against bacteremia	(16, 17)

*Legionella pneumophilia*	Gram−, intracell	Intranasal	Model of lung infection	**Protective**	Inhibition of intracellular replication of the pathogen and protection against bacteremia	(18, 19)

*Helicobacter pylori*	Gram−, extracell	Oral	Stomach infection/gastric mucosa infection	**Protective**	Induction of CXCL10 and reduction of bacterial burden in gastric mucosa	(20)

*Staphylococcus aureus*	Gram+, extracell	Intranasal	Model of lung infection	**Detrimental**	Exacerbated inflammatory cytokine production and leukocyte recruitment	(14, 21)

*Mycobacterium tuberculosis*	Intracell	Aerogenic	Model of lung infection	**Detrimental**	Immunosuppression (inhibition of IL-1 production and Th1 responses)	(22–24)

*Listeria monocytogenes*	Gram+, intracell	Tail vein injection	Model of systemic infection	**Detrimental**	Induction of apoptosis	(25, 26)

		Intraperitoneal	Model of systemic infection	**Detrimental**	Induction of apoptosis in the spleen and supression of IFN-γ production	(27, 28)

		Tail vein injection	Model of systemic infection	**Detrimental**	Suppression of IFNGR expression	(29)

		Intragastric	Model of gastrointestinal infection	**Protective**	Upregulation of protective cytokines limits hepatic inflammation	(28)
		Through food	Model of gastrointestinal infection	**No effect**		(30)

*Francisella tularensis* subspecies *tularensis*	Gram−, intracell	Intranasal	Model of tularemia	**Detrimental**	Inhibition of IL-17A	(31)

*Francisella tularensis s*ubspecies *novicida*	Gram−, intracell	Intradermal	Model of intradermal infection	**Detrimental**	Induction of macrophage death, inhibition of IL-17A and increased bacterial loads	(31, 32)

*Salmonela enterica* serovar Typhimurium	Gram−, intracell	Tail vein injection; intraperitoneal	Model of systemic infection	**Detrimental**	Enhancement of macrophage necroptosis and failure to control baterial burden	(33)

		Oral	Model of gastrointestinal infection	**Detrimental**	Immunosppression (inhibition of IL-1β, CXCl1 and CXCL2)	(34)

*Coxiella burnetii*	Gram−, intracell	Intratracheal	Model of lung infection	**Detrimental**	Promotion of dissemination	(35)

		Intratracheal infection with intraperitoneal rIFNα administration	Model of lung infection	**Detrimental**	Inhibition of inflammatory response in lungs	

		Intratracheal infection with intratracheal rIFNα administration	Model of lung infection	**Protective**	Reduction in bacterial dissemination	

Postinfluenzal bacterial pneumonia		Intratracheal; oropharyngeal aspiration	Model of lung infection	**Detrimental**	Attenuation of inflammatory response and leukocyte recruitment	(36–39)

## Type I IFN Induction by Bacteria

Type I IFNs were described more than a half century ago as products which are secreted by virus-infected cells and interfere with virus replication in autocrine and paracrine ways (40). It is now known that type I IFNs are cytokines produced in response to viral, bacterial, and fungal pathogens, as well as parasites. The effector mechanisms of type I IFNs mainly derive from products of genes which are transcriptionally regulated by type I IFN signaling. Type I IFNs induce hundreds of interferon-stimulated genes (ISGs) through activation of the homodimeric STAT1 and the heterotrimeric STAT1–STAT2–IRF9 (i.e., ISGF3) transcription factors (41). Bacteria trigger type I IFN production mostly following the recognition of bacterial nucleic acids or the Gram-negative cell wall component lipopolysaccharide (LPS) by innate immune receptors (12, 42). The induction mechanisms have been best studied for IFN-β which belongs together with IFNα4 to the first type I IFNs produced during infection and is the driver of other type I IFN genes (43, 44). The induction of IFN-β by bacterial DNA is complex and involves different pathways. The most common mode of IFN-β induction by bacterial DNA is through the cytosolic DNA sensor cyclic GMP-AMP synthase, as described for *Francisella novicida*, group B streptococcus (GBS) (*Streptococcus agalactiae*), *Legionella pneumophila, Listeria monocytogenes, Mycobacterium tuberculosis*, and *Neisseria gonorrhoeae* (45–51). The cytosolic DNA sensor Ifi204 (IFI16 in humans) contributes to IFN-β induction in the course of *F. novicida* and *L. monocytogenes* (48, 49). The signaling events downstream of these DNA sensors involve the STING–TBK1–IRF3 pathway driving the IFN-β gene transcription. *L. monocytogenes* can activate this pathway also independently of DNA sensing. This alternative mechanism is driven by binding of the bacterial product c-di-AMP to STING (51, 52). The recognition of bacterial DNA through unmethylated CpG motif-containing DNA by the endosomal Toll-like receptor 9 can also contribute to IFN-β induction, although the importance and relevance of this pathway in the context of the overall IFN-β production and host response have not been entirely clarified (42). Bacterial RNA has been recently established as another key IFN-β inducer. A short and highly conserved sequence found in the bacterial 23S rRNA is recognized by the mouse TLR13 leading to Myd88- and IRF5-dependent IFN-β induction (9, 53–56). Human cells employ TLR8 as IFN-β-inducing RNA sensor rather than TLR13 which is missing in humans (9, 53, 57–59). The precise nature of bacterial RNA triggering the human TLR8 remains to be identified.

IFN-β induction by LPS occurs after binding of this ligand to the TLR4 and the following internalization into the endosome (60). Subsequently, a TRIF-dependent activation of the kinase TBK1 causes phosphorylation of the transcription factor IRF3 to stimulate IFN-β gene transcription. Endosomal signaling has been also implicated in IFN-β induction by TLR2 in response to Gram-positive bacteria, although this mechanism appears to be restricted to specific immune cells and/or pathogens (61–63). The cytosolic receptors NOD1 and NOD2 were reported to trigger IFN-β production following infection with *Helicobacter pylori* and *M. tuberculosis*, respectively (20, 64). NOD2 engagement can induce IFN-β also in responses to *Staphylococcus aureus* (14).

While the pathways causing IFN-β induction by bacteria are relatively well understood, more studies are needed to assess the importance of individual pathways for the overall IFN-β production in whole organism rather than cells. One of the rare studies on this topic revealed that the dominant IFN-β-inducing pathway during infection with *Streptococcus pyogenes* (group A streptococcus) is the TLR13-mediated RNA recognition pathway (9).

Additional work is also needed to clarify the key IFN-β producing cells, as investigated during, e.g., *L. monocytogenes* infections (65–67) and the reported cell type-specific features of IFN-β induction (12, 15, 16).

## Tipping the Balance I: Benefits of Immunomodulatory Effects of Type I IFN Signaling During Bacterial Infections

Type I IFNs’ ability to stimulate immune responses against viruses has been established very early after their discovery but it soon became clear that these cytokines exhibit also immunosuppressive activities. The first evidence for such immunosuppressive activities was provided in a study showing that type I IFNs were able to reduce carrageenin-induced footpad swelling (68). Thus, type I IFNs are now regarded as immunomodulatory cytokines capable of enhancing or dampening the immune response, depending on the context. This ambiguousness contributes to the disparate and still incompletely understood roles of type I IFNs during bacterial infections. Importantly, no unifying principles have been found to date: neither the beneficial nor detrimental effects of type I IFN signaling correlate with the broad pathogen classification into Gram-positive and -negative, extra- and intracellular pathogens, or the route of infection (Table 1; Figure 1).

Immunosuppressive effects of type I IFN signaling are beneficial during infection with the Gram-positive largely extracellular human pathogen *S. pyogenes* (9). *S. pyogenes* is the causative agent of mild (e.g., pharyngitis and scarlet fever) but also invasive and life-threatening infections (e.g., cellulitis, necrotizing fasciitis, and streptococcal toxic shock syndrome). Mice deficient in the type I IFN receptor IFNAR1 are more susceptible to subcutaneous *S. pyogenes* infection, which is a relevant model of severe invasive infection of the soft tissue (15). Type I IFN signaling promotes resistance against *S. pyogenes* by suppressing the transcription of the *Il1b* gene (9). The absence of type I IFN signaling results in an unrestricted production of IL-1β thereby causing a lethal hyperinflammation and organ damage. Importantly, type I IFN signaling balances rather than prevents *Il1b* transcription so that a controlled and life-saving IL-1β production is achieved (9). The key IFN-β producer and effector cells in this infection model are both LysM+ and CD11c+ myeloid cells (9).

Immunomodulation by type I IFN signaling is protective during infection with the human Gram-positive extracellular pathogen GBS (16, 17). GBS is regarded as commensal microbe asymptomatically colonizing the skin and mucosal tissues of 30% people, yet it is the leading cause of severe neonatal pneumonia and meningitis in developed countries. The absence of IFNAR1 results in increased bacterial loads during both subcutaneous GBS infection of neonate mice and intravenous infection of adult animals (16, 17). Similarly, type I IFN signaling is protective against uncontrolled bacteremia during infection with the Gram-negative intracellular bacterium *L. pneumophila*, which is a frequent cause of the severe pneumonia, Legionnaire’s disease (18). Type I IFN signaling inhibits in a cell-autonomous way replication of *L. pneumophila* inside the infected cell (18, 19). The organismal physiology of the protective effects of type I IFN signaling during infection with GBS and *L. pneumophila* remains to be elucidated so that it is presently unclear whether immunosuppressive or immunostimulatory effects of type I IFNs drive the resistance against these two pathogens.

Stimulation of the immune response by type I IFN signaling is advantageous in defense against the Gram-negative pathogen *H. pylori* (20). *H. pylori* is a frequent cause of chronic gastritis and is associated with increased risk of gastric ulcers and stomach cancer. Deficiency in type I IFN signaling causes increased *H. pylori* loads in the stomach of orally infected mice. The lack of type I IFN responses is associated with decreased levels of the chemokine CXCL10 suggesting that type I IFNs promote defense against *H. pylori* by stimulating CXCL10-driven inflammation (20). Immunostimulatory effects of type I IFNs are beneficial also during gastric infection with the food-borne Gram-positive intracellular pathogen *L. monocytogenes* (28). *L. monocytogenes* infects the gastrointestinal tract, where it traverses the epithelial barrier and spreads into distant organs. Deficiency in type I IFN signaling results in an increased bacterial dissemination and is accompanied by diminished production of several pro-inflammatory cytokines, including TNF and IL-6 upon gastric infection using oral gavage (28). Interestingly, type I IFN signaling plays no role in an infection model using food contaminated with *L. monocytogenes* (30).

## Tipping the Balance II: Disadvantages of Immunomodulatory Effects of Type I IFN Signaling During Bacterial Infections

Immunosuppression by type I IFN signaling is detrimental during infection with the intracellular pathogen and causative agent of tuberculosis, *M. tuberculosis* (22–24). Type I IFN signaling-mediated inhibition of IL-1 cytokines during *M. tuberculosis* lung infection blunts the antimicrobial defense and results in increased local as well as systemic bacterial loads (24). The key type I IFN effector cells are transplantable inflammatory monocyte-macrophage cells and DCs (24). The precise mechanism of IL-1 inhibition by type I IFN signaling in this infection model is not resolved but includes both direct as well as indirect mechanisms (24). The indirect IL-1 inhibition appears to be mediated by the anti-inflammatory cytokine IL-10 which is known to be upregulated by type I IFNs (69). The importance of type I IFN signaling in *M. tuberculosis* infections is underlined by type I IFN signaling-associated gene expression pattern found in blood cells of patients with active tuberculosis (70). The detrimental function of type I IFN signaling during *M. tuberculosis* lung infection is converted into a protective one if IFN-γ signaling is missing (71). Under such conditions, type I IFNs inhibit the polarization of macrophages into infection-permissive alternatively activated macrophages.

Inhibition of immune response by type I IFNs is deleterious during infection with the facultative intracellular Gram-negative bacterium *F. novicida* (31). *F. novicida* is a subspecies of *Francisella tularensis* which infects humans through the skin or aerosol droplets and causes ulceroglandular or pneumonic tularemia, respectively. IFNAR1-deficient mice infected intradermally with *F. novicida* respond by an increased IL-17 production compared to WT animals and, correspondingly, are more resistant against infection (31). Similar increase in resistance is also observed during lung infection with *F. tularensis* (31). The key IL-17 producers during *F. novicida* infection are IL-17A+ γδ T cells which show enhanced expansion in the absence of type I IFN signaling.

Interferon-β exacerbates infection with *S. typhimurium* by reducing the ability of the host to launch a complete immune response (34). *S. typhimurium* is Gram-negative intracellular pathogen associated with gastroenteritis in humans and a severe disease resembling typhoid fever in mice. Mice deficient in IFN-β are more resistant against oral infection with *S. typhimurium* and display enhanced expression of IL-1β and the neutrophil chemoattractants, CXCL1 and CXCL2 (34). These changes are attributable to IFN-β-mediated inhibition of these genes in macrophages and are independent of *S. typhimurium*-induced macrophage death.

*Listeria monocytogenes*-induced type I IFN signaling downregulates the expression of both type II IFN receptor subunits, IFNGR1 and IFNGR2, thereby decreasing the responsiveness of macrophages and DCs to IFN-γ (29). The suppression of the IFN-γ signaling results in an increased susceptibility to *L. monocytogenes* infection. Increased susceptibility to *L. monocytogenes* infection is also caused by type I IFN-mediated induction of anti-inflammatory IL-10 (72).

Immunosuppressive effects of type I IFNs are harmful during postinfluenzal bacterial pneumonia (36–39). The immune response during a secondary postinfluenzal infection with the Gram-positive extracellular pathogen *S. pneumoniae*, a key causative agent of pneumonia, is impaired. This is caused by the ability of type I IFNs to suppress production of the neutrophil chemoattractants CXCL1 and CXCL2, the macrophage chemoattractant CCL2, and the inflammation-promoting cytokine IL-17 (36–38). The resulting reduction of leukocyte infiltration in the lung diminishes the capability of the host to control bacterial growth. Similar alterations in the immune response appear to be responsible for the increased susceptibility of mice to postinfluenzal infection with *S. aureus* and *Pseudomonas aeruginosa* (39). The mechanisms of the immunosuppressive effects of type I IFN signaling during postinfluenzal bacterial infection are not well understood but they might act downstream of the type I IFN-mediated inhibition of IL-1 cytokines.

Enhancement of the inflammatory response is associated with detrimental effects of type I IFN signaling during lung infection with the Gram-positive extracellular pathogen *S. aureus* (21). IFNAR1-deficient mice exhibit a lower TNF and IL-6 production and decreased leukocyte infiltration in the lung compared to WT animals suggesting that type I IFN signaling causes an exacerbated tissue damage (21). The pathogenicity of *S. aureus* strains correlates with the levels of type I IFNs induced during infection with differently virulent strains (14).

## Tipping the Balance III: Regulation of Tissue and Cell Integrity by Type I IFNs During Bacterial Infections

Type I IFN signaling plays an indispensable role in the preservation of the epithelial barrier and epithelial integrity during lung infection with *S. pneumoniae* (10, 11). Type I IFN signaling promotes the maintenance of lung epithelial tight junctions during *S. pneumoniae* infection thereby reducing the passage of the pathogen from alveoli into the lung parenchyma (10). IFNAR1-deficient mice display increased permeability of the lung epithelium and enhanced invasiveness of *S. pneumoniae* infection associated with higher bacterial burden in distant organs. Type I IFN signaling protects the barrier function of the lung during *S. pneumoniae* infection also by promoting survival of the alveolar epithelial type II cells as revealed by IFNAR1 deletion specifically in this subtype of the barrier epithelium (11).

A common detrimental effect of type I IFN signaling during bacterial infections is the induction of various types of leukocyte cell death. Type I IFN-facilitated apoptosis of macrophages and lymphocytes appears to contribute to the increased susceptibility of WT mice compared to IFNAR1-deficient animals to intravenous and intraperitoneal infection with *L. monocytogenes* (25–27, 73). Type I IFN-facilitated death of macrophages is associated also with harmful effects of type I IFNs during infection with *F. novicida* and *S. typhimurium* (32, 33). *F. novicida* promotes macrophage death by type I IFN-mediated inflammasome-activation whereas *S. typhimurium* employs type I IFN induction to stimulate RIP-dependent macrophage necroptosis.

## Conclusion and Future Directions

Ample evidence exists for the pivotal role of type I IFNs in regulation of defense against bacterial pathogens. The complex and often disparate effects of type I IFNs on the outcome of different bacterial infections provide chances to exploit type I IFNs and their inducers as well as effectors for adjuvant therapies tailored to specific infectious diseases. A prerequisite for the development of such therapies is a detailed understanding of the molecular, cellular, and organismal physiology of type IFNs in the course of bacterial infections. The following topics appear particularly important since they represent rather underexplored yet critically important areas.

### Pathogen Species

The inconsistent roles of type I IFNs during infections with different bacteria remain a significant and challenging topic in the current research. Only few common principles of type I IFN action have been found to date. They include the cell death-promoting effects of type I IFNs which contribute mostly to detrimental functions of type I IFN signaling. Another frequent observation is the suppression of IL-1β and neutrophil chemoattractants—these effects are, however, associated with both beneficial and harmful consequences for the infection outcome. Future studies employing pathogens which have not yet been analyzed in detail, such as *Klebsiella pneumoniae*, uropathogenic *E. coli*, or *Clostridium difficile*, might reveal novel common principles.

### Infection Route and Tissue-Specific Features of Type I IFN Signaling

The complexity of type I IFN function in bacterial infections is further increased by the distinct effects of type I IFNs in response to the same but differently administered pathogen. Type I IFNs are harmful followed intraperitoneal or intravenous infection with *L. monocytogenes* but protective in a physiologically more relevant intragastric infection (25–28). In contrast, type I IFN signaling has no impact on the overall outcome of *L. monocytogenes* infection after ingesting pathogen-contaminated food (30). These observations suggest that type I IFN signaling has, with regard to bacterial infections, distinct functions in different tissues/organs. This implication is supported by a recent study showing that exogenous type I IFN has, depending on the site of administration, disparate effects on the course of lung infection with *Coxiella burnetii* (35). Infections with *C. burnetii*, a Gram-negative intracellular bacterium, in humans occur after inhalation of bacteria and result in Q fever which can develop into an atypical pneumonia. Lung infection with *C. burnetii* in mice has a more severe course in WT compared to IFNAR1-deficient animals indicating that type I IFN signaling is disadvantageous in this infection model (35). Consistently, intraperitoneally administered type I IFN exacerbates *C. burnetii* infection. However, intratracheal delivery of type I IFN ameliorates the course of *C. burnetii* infection. The mechanisms of these distinct effects of type I IFN signaling in different tissues remain to be elucidated. Future studies should investigate other pathogens known of using various routes of infection and focus on physiologically most relevant routes.

### Most Significant Type I IFN Inducers and Effectors

Modulation of type I IFN production during bacterial infection might represent a powerful approach in therapy of infectious diseases. Therapeutic targeting of type I IFN production requires the knowledge of the most important type I IFN-inducing pathway in a given infection. As most bacterial pathogens employ more than one pathway to stimulate type I IFN production, future efforts should focus on the identification of the most crucial bacterial and cellular components involved in type I IFN induction. These studies will need to use a combination of bacterial and animal host genetics for functional assessment and suitable reporter as well as imaging systems for type I IFN detection *in vivo*. On the effector side, recent studies provided a number of novel type I IFN-induced factors which interfere with pathogen replication and survival. Notably, various type I IFN-inducible small GTP-binding proteins have recently been showed to significantly contribute to the effects of type I IFNs [e.g., Ref. (74–77)]. These and yet to be discovered effectors represent potential targets for therapeutic intervention.

### Human versus Mouse Systems

While many host defense mechanisms are well conserved among mice and men, important differences exist. For example, the mouse type I IFN inducer TLR13 is not expressed in humans (9, 53). Conversely, the human but not mouse TLR8 appears to be involved in type I IFN induction by bacterial RNA (59). Some antimicrobial functions of human neutrophils are enhanced by type I IFNs (78) whereas such stimulatory effects have so far not been described in mouse neutrophils. Future work should put more emphasis on studies of common and distinct features of type I IFN functions in bacterial infections.

## Author Contributions

All authors listed have made substantial, direct, and intellectual contribution to the work and approved it for publication.

## Conflict of Interest Statement

The authors declare that the research was conducted in the absence of any commercial or financial relationships that could be construed as a potential conflict of interest.
